# Modulation of Adenosine Receptors and Antioxidative Effect of Beer Extracts in in Vitro Models

**DOI:** 10.3390/nu11061258

**Published:** 2019-06-03

**Authors:** Patricia Alonso-Andrés, Mairena Martín, José Luis Albasanz

**Affiliations:** Department of Inorganic and Organic Chemistry and Biochemistry, Faculty of Chemical and Technological Sciences, School of Medicine of Ciudad Real, Regional Center of Biomedical Research (CRIB), University of Castilla-La Mancha (UCLM), 13071 Ciudad Real, Spain; patrialan22@gmail.com (P.A.-A.); mairena.martin@uclm.es (M.M.)

**Keywords:** adenosine receptors, oxidative stress, beer

## Abstract

The fight against neurodegenerative diseases is promoting the searching of nutrients, preferably of wide consumption, with proven effects on health. Beer is widely consumed and has potential benefits on health. In this work, three different extracts from dark beer (DB), non-alcoholic beer (NAB), and lager beer (LB) were assayed at 30 min and 24 h in rat C6 glioma and human SH-SY5Y neuroblastoma cells in order to study their possible protective effects. Cell viability and adenosine A_1_, A_2A_, A_2B_, and A_3_ receptor gene expression and protein levels were measured in control cells and in cells challenged with hydrogen peroxide as an oxidant stressor. Among the three extracts analyzed, DB showed a greater protective effect against H_2_O_2_-induced oxidative stress and cell death. Moreover, a higher A_1_ receptor level was also induced by this extract. Interestingly, A_1_ receptor level was also increased by NAB and LB extracts, but to a lower extent, and the protective effect of these extracts against H_2_O_2_ was lower. This possible correlation between protection and A_1_ receptor level was observed at 24 h in both C6 and SH-SY5Y cells. In summary, different beer extracts modulate, to a different degree, adenosine receptors expression and protect both glioma and neuroblastoma cells from oxidative stress.

## 1. Introduction

The development of neurodegenerative diseases that include dementia, as in Alzheimer’s disease (AD), has been partially associated with nutritional factors [1,2]. There are several compounds taken from the diet with protective effect against dementia, such as antioxidants (some vitamins) or flavonoids [3], that would protect other beneficial compounds against oxidation (e.g., polyunsaturated fatty acids, PUFAs) [4]. Therefore, the beneficial properties of low alcoholic graduation like that of beer could be associated with antioxidant strength [5], due to the presence of natural compounds such as xanthohumol [6] and resveratrol [7].

Beer is one of the most consumed drinks, after water and tea [8]. This beverage has as ingredients water, malt, non-malted cereals, hops, and yeast (which is responsible for the fermentation process) [9]. Moreover, there are more than 800 organic compounds present in the raw materials or produced by fermentation that act as flavorings [10]. Therefore, it is very difficult to generalize about beer composition, because each beer contains a wide range of compounds, depending on the raw materials used for its production and the brewing process [11].

In addition, beer contains several polyphenols, including flavonoids and phenolic acids, that are responsible for its organoleptic properties, such as flavor, and appearance

Due to its composition, including polyphenols (e.g., flavonoids and phenolic acids), several beneficial effects on health have been associated to beer, such as a diuretic effect, a high nutritional contribution (vitamins, fibers, and antioxidants), and hunger stimulation in aging [5,11,12,13].

Neurological effects are observed after flavonoids (e.g., sophorine) intake, associated with the prevention of neurological diseases such as Parkinson’s disease (PD) [14,15]. Beer is able to contribute to the increase of silicon quantity by limiting aluminum availability, considered a neurotoxin in AD, and by normalizing antioxidant enzymes gene expression and tumoral necrosis factor levels [16]. Other studies propose a moderate intake of beer in order to decrease beta-amyloid peptide aggregation [17], activate microglia, and prevent inflammation [18]. Oxidative stress is a factor involved in aging processes [19]. In neurodegenerative diseases such as AD, oxidative stress is enhanced during the development of the disease [20]. Several studies have tested compounds for their ability to improve cognitive capacities in AD models, reducing oxidative stress and other associated processes. For example, ellagic acid is able to reduce oxidative stress in SH-SY5Y cells and in rats with streptozotocin-induced Sporadic Alzheimer Disease [21]. In addition, hydroxyurea attenuates oxidative stress in hippocampal neurons in rat, improving spatial memory in mice model of AD [22].

Several molecular targets have been described for polyphenols, specifically resveratrol, as estrogen receptor α, cyclooxygenase 1, NF-ΚB, and adenosine receptors [23,24]. Adenosine is a ubiquitous and homeostatic molecule considered a neuromodulator that exerts its effects through four specific G protein-coupled receptors: A_1_, A_2A_, A_2B_, and A_3_ [25]. It has been suggested that adenosine has a role in neurodegenerative diseases, including AD [26]. In fact, A_1_ and A_2A_ receptors are increased in the frontal cortex of AD patients [27], while several studies claim that caffeine intake, a potent antagonist of adenosine A_2A_ receptors, improves cognitive capacities in AD patients [28,29]. Adenosine regulates neuroplasticity, cognitive and motor function, and emotion behaviors and it is related to many cerebral dysfunctions in human diseases [30,31]. Additionally, adenosine receptors function and level have been involved in neuroprotective and antitumoral mechanisms in C6 [32,33,34,35,36,37] and SH-SY5Y cells [32,38,39,40,41].

It has been described that some polyphenols acting through adenosine receptors have an important implication in many several physiological processes. For example, the topic application of trans-resveratrol in rats is able to reduce ocular hypertension, in which adenosine A_1_ receptors are involved [42]. We have recently described the relation between adenosine receptors and polyphenols (resveratrol) in SAMP8 mice, suggesting the role of resveratrol through adenosine receptors and a possible participation in neuroprotective effects in neurodegenerative diseases [43]. Moreover, we have recently demonstrated that resveratrol, present in beer and wine, is able to modulate adenosine receptors by acting as a non-selective agonist of adenosine receptors in C6 glioma cells [24].

Therefore, the aim of the present work was to study the potential antioxidant protective effect and the possible modulation of adenosine receptors by three different commercial beer extracts (dark, non-alcoholic, and lager beers). To this end, two cellular models were used, rat C6 glioma and human SH-SY5Y neuroblastoma cells, which endogenously express adenosine receptors.

## 2. Materials and Methods

### 2.1. Materials

[^3^H]-DPCPX (8-Cyclopentyl-1,3-dipropylxanthine) (118 Ci/mmol) and [^3^H]-ZM 241385 ([2-^3^H](4-(2-[7-amino-2-(2-furyl) [1,2,4] triazolo [2,3-α] [1,3,5] triazin-5-ylamino] ethyl) phenol) 27.4 Ci/mmol) were purchased from Amersham Biosciences (Buckinghamshire, UK). Theophylline and hydrogen peroxide were acquired from Sigma Aldrich (Madrid, Spain). CPA (N^6^-Cyclopentyladenosine) was obtained from Tocris (Madrid, Spain). The Liquid scintillation cocktail was supplied by Perkin Elmer (Boston, MA, USA). Additional reagents were of analytical grade.

### 2.2. Cell Culture

Rat C6 glioma and human SH-SY5Y neuroblastoma cells were obtained from the American Type Culture Collection (ATCC). C6 cells were grown as described elsewhere [44]. For C6, Dulbecco’s modified Eagle’s medium was supplemented with 10% fetal bovine decomplemented serum (PAA, Parching, Austria), 2 mM L-glutamine, 1% non-essential amino acids, 1% antibiotic-antimycotic, and 50 µg/mL gentamicin (Gibco, Waltham, MA, USA). SH-SY5Y were grown in DMEM supplemented with 10% fetal bovine serum and 1% antibiotic–antimycotic [39]. Both cell types were grown in a humidified atmosphere of 95% air and 5% CO_2_ at 37 °C. C6 and SH-SY5Y cells were subcultured in 10 mL Petri dish (Nunc, Roskilde, Denmark). At confluence, C6 were detached by mechanical action, whereas SH-SY5Y were detached by trypsin (Tryple Express, Gibco, USA). In both cases, the cells were resuspended in complete growth medium and plated in 24- or 96-well dished (Nunc, Roskilde, Denmark) as necessary.

### 2.3. Cell Viability Assay

Cell viability was determined using an in vitro colorimetric assay kit based on the reduction of tetrazolium salt (XTT) converted to formazan in the presence of an electron-coupling agent, purchased from Roche (Cell Proliferation Kit II, XTT). C6 and SH-SY5Y cells were seeded (10^4^ cells per well) in 96-well dishes and exposed to beer extracts and/or an oxidant agent (50 µM H_2_O_2_) for 30 min or 24 h. The cells were incubated with the XTT solution for 30 min at 37 °C. The cleavage of XTT to form an orange formazan dye by viable cells was monitored by reading the absorbance at 475 and 690 nm, according to the manufacturer’s protocol (Cell Proliferation Kit II, XTT, Roche, Mannheim, Germany).

### 2.4. Beer Extracts Preparation

Three extracts were obtained from three different commercial brands of dark, non-alcoholic, and lager beers. A volume of 500 µL of beer was concentrated by SpeedVac, totally losing its alcohol content, and resuspended in 150 µL of cell culture medium before treatments (50 µL, 30 min, or 24 h).

### 2.5. Total RNA Isolation and Preparation of cDNA

Total RNA was extracted from the cells using an ABI 6100 Nucleic Acid PrepStation and chemicals according to the manufacturer’s protocol (Applied Biosystems, Foster City, CA, USA). The ratio of A_260_/A_280_ (purity of RNA) was in the range 1.8–2.0. RNA concentrations were determined from the absorbance A_260_. RNA was isolated and stored at −80 °C. One microgram of total RNA was reverse-transcribed using the Applied Biosystems’ High-Capacity cDNA Archive Kit.

### 2.6. Quantitative Real Time RT-PCR Analysis

To assess relative gene expression in C6 glioma and SH-SY5Y neuroblastoma cells, quantitative real time RT-PCR analysis was performed with an Applied Biosystems Prism 7500 Fast Sequence Detection System, using TaqMan^®^ Universal PCR Master Mix according to the manufacturer’s specifications (Applied Biosystems, Foster City, CA, USA). The validated TaqMan^®^ probes and primers for A_1_ (Hs00181231_m1), A_2A_ (Rn 00583935_m1, Hs00169123_m1), A_2B_ (Rn00567697_m1, Hs00386497_m1), A_3_ (Rn00563680_m1), and β-actin (Rn00667869_m1, Hs99999903_m1) were assay-on-demand gene expression products (Applied Biosystems). The TaqMan^®^ Gene Expression Assays have an efficiency of 1.0, which means the doubling of the PCR product in every cycle is guaranteed. The TaqMan^®^ primer and probe sequences are packaged together in a 20X solution. The sequences are proprietary, so they are not available. The gene-specific probes were labelled using the reporter dye FAM. A non-fluorescent quencher and the minor groove binder were linked at the 3′ end of the probes as quenchers. The thermal cycler conditions were as follows: 20 s at 95 °C, followed by two steps of PCR for 40 cycles at 95 °C for 3 s, followed by 60 °C for 30 s. The levels of RNA expression were determined using the 7500 Fast System SDS software version 1.3.1 (Applied Biosystems, Foster City, CA, USA) according to the 2^−ΔΔCt^ method. Then, the gene expression results were normalized to endogenous control β-actin relative to a calibrator, consisting of the mean expression level of the receptor gene as follows: 2^−ΔΔCt^ = 2 ^−((Ct receptor gene − Ct actin) sample − (Ct receptor gene − Ct actin) calibrator)^. β-actin is an appropriated endogenous control, as its expression did not change after beer and oxidant stress treatments. The results are from three or four independent assays, performed in different plates, each using different cDNAs from the cultures analyzed, and were averaged to produce a single mean value for each mRNA.

### 2.7. Radioligand Binding Assays

#### 2.7.1. Adenosine A_1_ Receptor in C6 and SH-SY5Y Cells

Adenosine A_1_ receptor (A_1_R) in C6 and SH-SY5Y cells was quantified by radioligand binding assay using [^3^H]-DPCPX, a selective A_1_R antagonist, as a radioligand (Amersham Biosciences, Buckinghamshire, UK), as described previously [44]. [^3^H]-DPCPX was used at the saturating concentration of 20 nM, and 4 mM CPA, a selective agonist of A_1_R, was employed to obtain non-specific binding. The cells were previously treated with 5 U/mL adenosine deaminase (ADA) for 30 min at 37 °C in order to remove endogenous adenosine. Then, the cells were incubated with [^3^H]-DPCPX for 2 h at 25 °C without shaking. After this time, the cells were washed with ice-cold culture medium, lysed with 0.2% SDS, and transferred to vials to count radioactivity. Two wells from each plate were employed for protein content measurement. Radioactivity measurements in vials were performed in the Microbeta Trilux Jet scintillation counter (Perkin Elmer) using Optiphase HiSafe scintillation liquid (Perkin Elmer).

#### 2.7.2. Adenosine A_2A_ Receptor in SH-SY5Y Cells

Adenosine A_2A_ receptor (A_2A_R) was assayed in intact SH-SY5Y cells by a radioligand binding assay using [^3^H] ZM241385 as a specific radioligand. Cells grown in 24-well plates were incubated with a saturating concentration of 40 nM [^3^H]-ZM241385, as described earlier [45]. ADA (5 U/mL) was used to remove endogenous adenosine. Theophylline (5 mM) was used to obtain non-specific binding. After 2 h at 25 °C, the cells were washed with ice-cold culture medium, lysed with 0.2% SDS, and transferred to vials to count radioactivity. Two wells from each plate were employed for protein content measurement. Radioactivity measurements in vials were performed in the Microbeta Trilux Jet scintillation counter (Perkin Elmer) using Optiphase HiSafe scintillation liquid (Perkin Elmer).

### 2.8. Protein Determination

Protein concentration was measured by the method of Lowry, using serum albumin as a standard [46].

### 2.9. Statistical and Data Analysis

Data are means ± SEM. Statistical analysis was performed using Student’s *t*-test and one-way ANOVA (Analysis of Variance) followed by Tukey’s post-hoc test for multiple-comparison analysis. Differences between mean values were considered statistically significant at *p* < 0.05 using GraphPad Prism 7.0 (GraphPad Software, San Diego, CA, USA).

## 3. Results

### 3.1. Effect of Beer Extracts on C6 and SH-SY5Y Cell Viability

**Figure 1:**
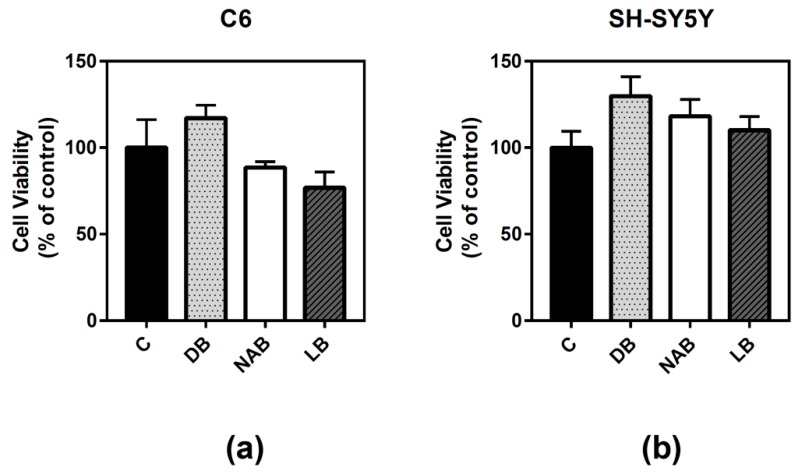
Effect of different beer extracts on C6 and SH-SY5Y cell viability. Dark beer (DB), non-alcoholic beer (NAB), and lager beer (LB) extracts were added to C6 (**a**) and SH-SY5Y (**b**) cells for 24 h. Data are means ± SEM of, at least, three independent assays. Differences were not statistically significant according to Student’s *t* test or one-way ANOVA.

Cell viability was studied in two different models, rat astroglioma C6 and human neuroblastoma SH-SY5Y cells, in order to compare possible differences between these cell types. The cells were treated for 24 h with three different beer extracts corresponding to dark beer (DB), non-alcoholic beer (NAB), and lager beer (LB). The procedure to obtain these extracts is described in Materials and Methods. C6 (Figure 1a) and SH-SY5Y (Figure 1b) cells viability was not significantly altered by the treatment with the beer extracts.

### 3.2. Effect of Beer Extracts on Adenosine Receptor Gene Expression in C6 and SH-SY5Y Cells

**Figure 2:**
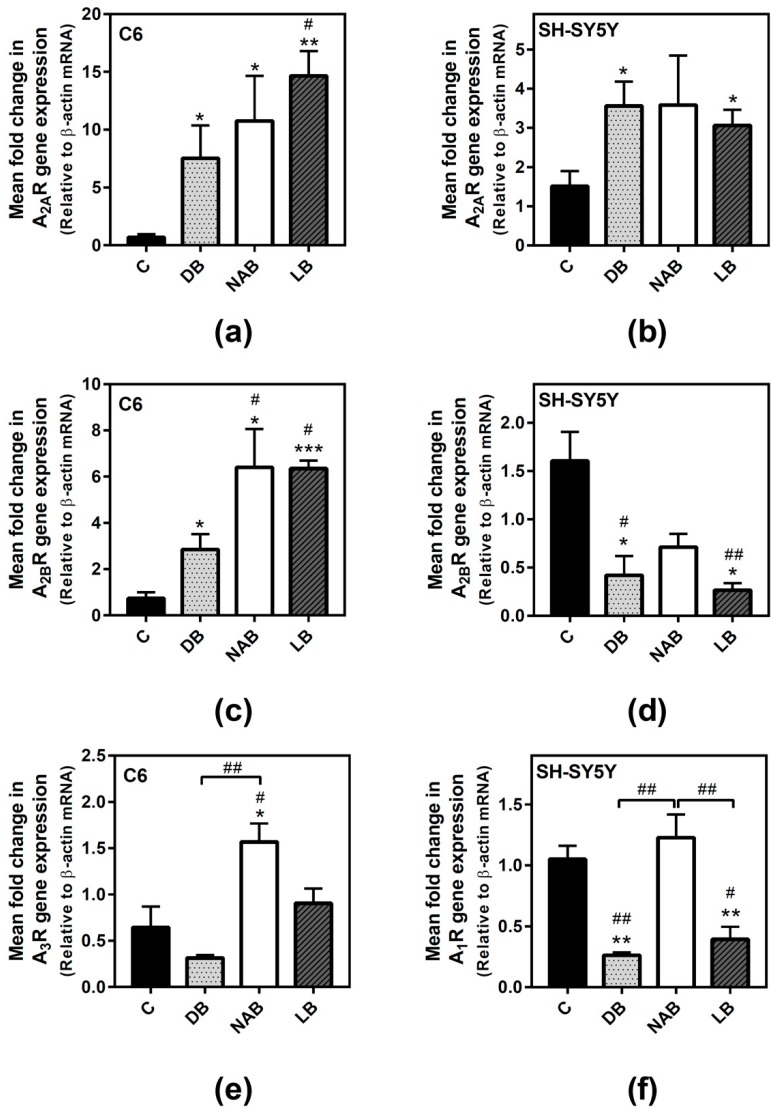

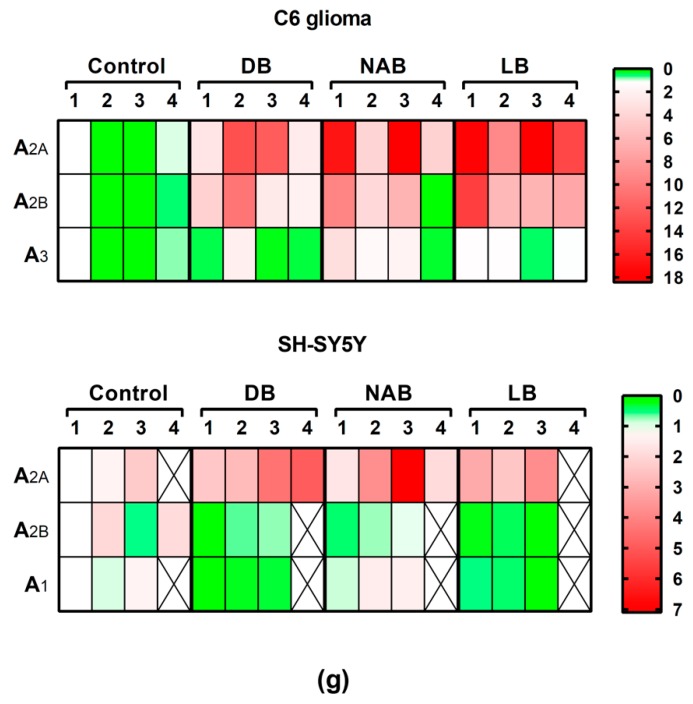
Adenosine receptors gene expression assayed by quantitative real-time RT-PCR in C6 and SH-SY5Y cells. After total RNA isolation, A_2A_ (panels a and b), A_2B_ (panels c and d), A_3_ (panel e), and A_1_ (panel f) receptor gene expression levels were detected by using TaqMan universal PCR following the protocol indicated in “Materials and Methods”. β-actin was used as an endogenous control in all assays. The treatments were performed for 24 h with DB, NAB, and LB extracts in C6 (**a**,**c**,**e**) and SH-SY5Y (**b**,**d**,**f**) cells. (**g**) Heatmap of gene expression levels obtained from the analyzed samples. Data are means ± SEM of, at least, three independent assays; * *p* < 0.05, ** *p* < 0.01, and *** *p* < 0.001, significantly different from their corresponding control (C) according to Student’s *t* test; # *p* < 0.05, and ## *p* < 0.01, significantly different from control (C) or considered bars according to one-way ANOVA test.

After RNA isolation, real-time PCR assays were performed in order to know whether the beer extracts modulated adenosine receptors’ gene expression. The treatments with DB (*p* < 0.05), NAB (*p* < 0.05), and LB (*p* < 0.01) significantly increased A_2A_ gene expression (Figure 2a) in C6 cells. This higher expression was also detected in SH-SY5Y cells exposed to DB (*p* < 0.05) or LB (*p* < 0.05) (Figure 2b). A_2B_ expression was also increased in C6 cells with the exposure to the beer extracts, being greater in cells exposed to NAB (*p* < 0.05) and LB (*p* < 0.001) than in those exposed to DB (*p* < 0.05) (Figure 2c). On the contrary, DB and LB extracts significantly (*p* < 0.05) decreased A_2B_ gene expression in the cells, and a lower but not statistically significant expression was observed after treatment with NAB treatment (Figure 2d). A_3_ gene expression was only significantly increased by NAB (*p* < 0.05) in C6 cells (Figure 2e). In SH-SY5Y cells, A_1_ gene expression was decreased by the exposure to DB and LB (*p* < 0.01) (Figure 2f). Unfortunately, we could not amplify neither A_1_ expression in C6 nor A_3_ expression in SH-SY5Y cells under our experimental conditions.

### 3.3. Effect of Beer Extracts on Adenosine A_1_ and A_2A_ Receptors Levels

**Figure 3:**
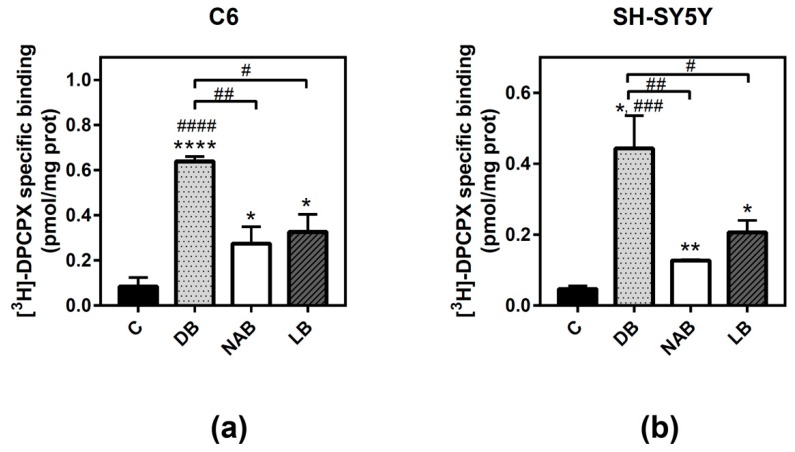
Analysis of adenosine A_1_ receptors by radioligand binding assay in C6 and SH-SY5Y cells. Specific binding to A_1_ receptors was determined in C6 (panel **a**) or SH-SY5Y (panel **b**) intact cells at saturating concentration (20 nM) of [^3^H]-DPCPX, as described in “Materials and Methods”. Nonspecific binding was determined in the presence of 4 mM CPA. The treatments were performed for 24 h with DB, NAB, and LB extracts. Data are means ± SEM of, at least, three independent assays; * *p* < 0.05, ** *p* < 0.01, and **** *p* < 0.0001, significantly different from their corresponding control (C) according to Student’s *t* test; # *p* < 0.05, ## *p* < 0.01, ### *p* < 0.001, and #### *p* < 0.0001, significantly different from control (C) or considered bars according to one-way ANOVA test.

As one of the main adenosine receptors expressed and modulated in these cells according to our previous results are A_1_ receptors, the next step was to quantify these receptors in both C6 and SH-SY5Y cells by radioligand binding assays. All extracts of beer significantly increased adenosine A_1_ receptor level in both C6 (Figure 3a) and SH-SY5Y (Figure 3b) intact cells after 24 h of exposure. However, DB produced the highest and most significant increase (C6: *p* < 0.0001, and SH-SY5Y: *p* < 0.05) of these receptors, followed by LB (C6: *p* < 0.05, and SH-SY5Y: *p* < 0.05) and NAB (C6: *p* < 0.05, and SH-SY5Y: *p* < 0.01).

**Figure 4:**
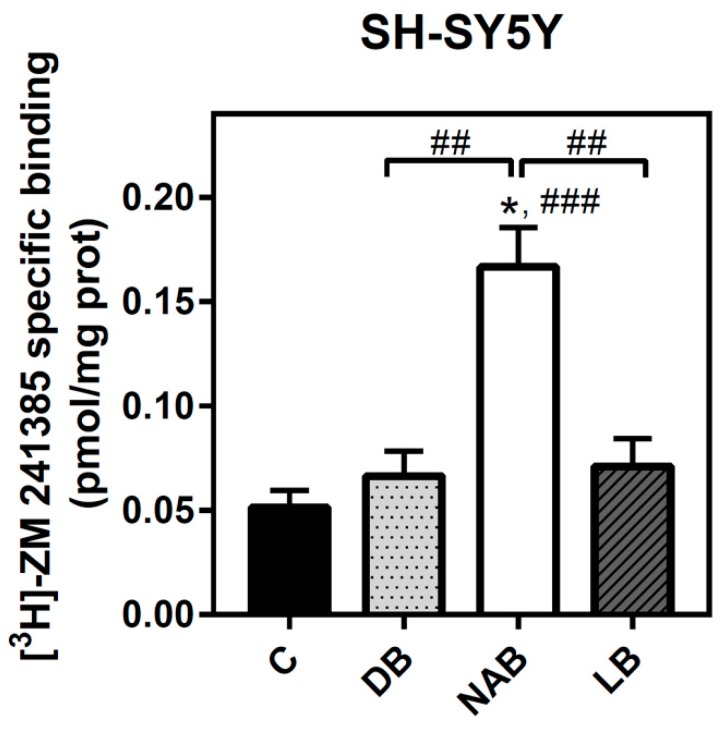
Analysis of adenosine A_2A_ receptors by radioligand binding assay in SH-SY5Y cells. Specific binding to A_2A_ receptor was determined in intact cells at saturating concentration (40 nM) of [^3^H]-ZM 241385, as described in “Materials and Methods”. Nonspecific binding was determined in the presence of 12 mM theophylline. Treatments were performed for 24 h with DB, NAB, and LB extracts in SH-SY5Y cells. Data are means ± SEM of, at least, three independent assays; * *p* < 0.05 significantly different from their corresponding control (C) according to Student’s *t* test; ## *p* < 0.01 and ### *p* < 0.001, significantly different from control (C) or considered bars according to one-way ANOVA test.

Adenosine A_2A_ receptor was also quantified in SH-SY5Y intact cells. The NAB extract was the only one able to significantly increase the level of these receptors after 24 h of exposure (Figure 4).

### 3.4. Antioxidant Ability of Beer Extracts in C6 and SH-SY5Y Cells

**Figure 5:**
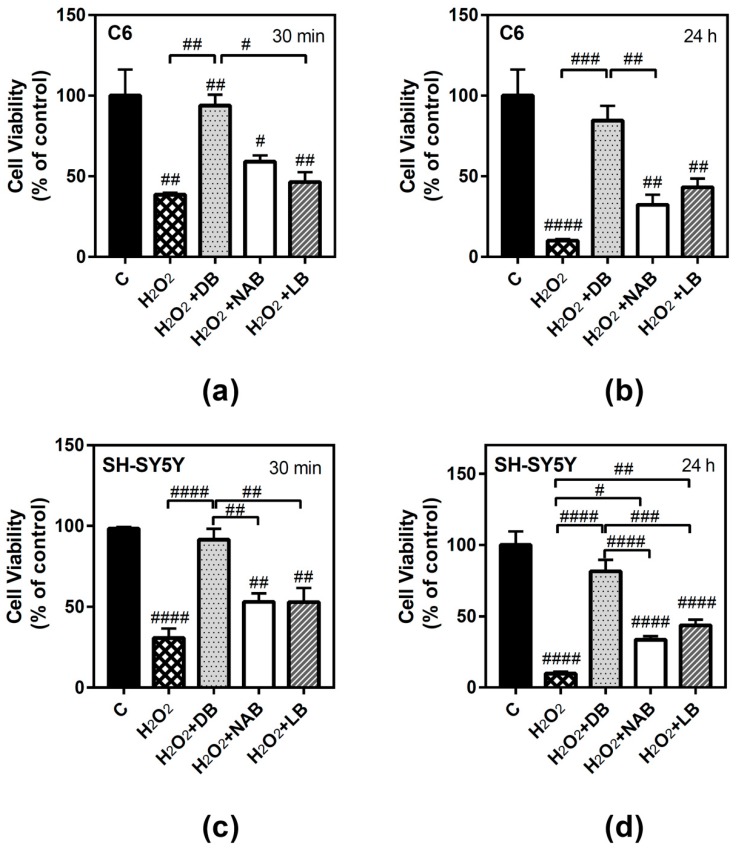

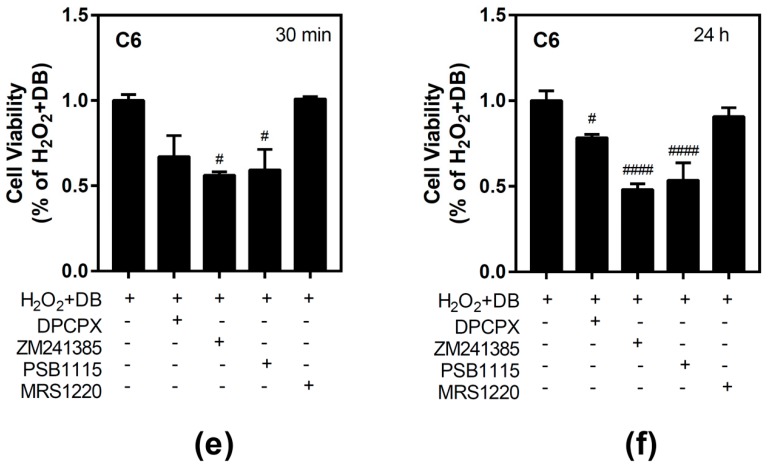
Effect of different beer extracts on cell viability in the presence of the oxidative stress induced by hydrogen peroxide (H_2_O_2_) in C6 and SH-SY5Y. Treatments were performed for 30 min and 24 h with 50 µM H_2_O_2_ and DB, NAB, and LB extracts in C6 (panels **a** and **b**) and SH-SY5Y (panels **c** and **d**) cells. Effect of 10 µM DPCPX, 100 µM ZM241385, 100 µM PSB1115, and 10 µM MRS1220, specific adenosine A_1_, A_2A_, A_2B_, and A_3_ receptor antagonists, respectively, in the presence of 50 µM H_2_O_2_ and DB (panel **e** and **f**). Data are means ± SEM of, at least, three independent assays; # *p* < 0.05, ## *p* < 0.01, ### *p* < 0.001, and #### *p* < 0.0001, significantly different from control (C) or considered bars according to one-way ANOVA test.

The antioxidant ability of beers was proved for short (30 min) and long (24 h) times. To this end, hydrogen peroxide (50 µM H_2_O_2_) treatment was chosen as the oxidative stress agent. One-way ANOVA analysis revealed that this compound significantly reduced C6 cells viability at both times of treatment (Figure 5a, *p* < 0.01 and 5b, *p* < 0.0001), and the effect of cell death was more accentuated at 24 h. Although similar profiles were observed in all cases, the antioxidant ability (i.e., the reduction of cell viability decrease elicited by H_2_O_2_) was more potent and significant for DB extracts at 24 h (Figure 5b, *p* < 0.001) and at 30 min (Figure 5a, *p* < 0.01).

Similarly, H_2_O_2_ also reduced SH-SY5Y cells viability at 30 min (Figure 5c, *p* < 0.0001) and 24 h (Figure 5d, *p* < 0.0001), and the effect of cell death was more accentuated at 24 h. At 30 min, DB extract caused again the most potent antioxidative effect (*p* < 0.0001) (Figure 5c). Increasing the exposure time to 24 h, a similar profile was observed: DB (*p* < 0.0001), LB (*p* < 0.01), and NAB (*p* < 0.05) extracts recovered cell viability with respect to hydrogen peroxide (Figure 5d), showing again an antioxidant and protective effect. Interestingly, DB extract showed the highest antioxidant ability at both times and for both cell types analyzed.

In agreement with a possible participation of adenosine receptors in the protective effect of DB, the presence of several adenosine receptors antagonists significantly reduced cell viability measured in the presence of H_2_O_2_ and DB at 30 min (Figure 5e) and at 24 h (Figure 5f) in C6 glioma cells.

### 3.5. Effect of Beer Extracts and Oxidative Stress on Adenosine Receptors Gene Expression in C6 and SH-SY5Y Cells

**Figure 6:**
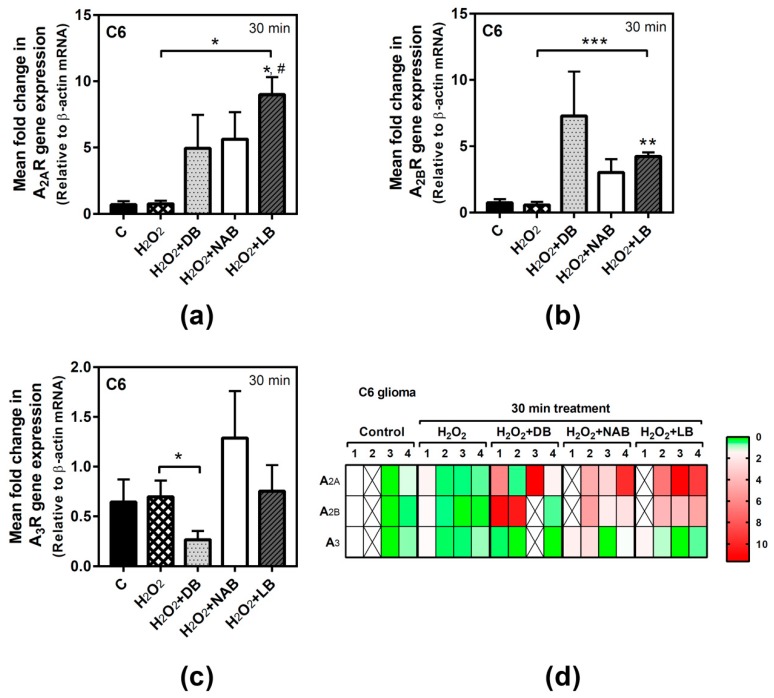
Adenosine receptors gene expression assayed by quantitative real-time RT-PCR in C6 cells. After total RNA isolation, A_2A_ (panel **a**), A_2B_ (panel **b**), and A_3_ (panel **c**) receptor gene expression levels were detected by TaqMan universal PCR following the protocol indicated in “Materials and Methods”. β-actin was used as an endogenous control in all assays. Treatments were performed for 30 min with 50 µM hydrogen peroxide (H_2_O_2_) and DB, NAB, and LB extracts in C6 cells. (**d**) Heatmap of gene expression levels obtained in the analyzed samples. Data are means ± SEM of, at least, three independent assays; * *p* < 0.05, ** *p* < 0.01, and *** *p* < 0.001, significantly different from their corresponding control (C) or considered bars according to Student’s *t* test; # *p* < 0.05 significantly different from control (C) according to one-way ANOVA test.

In order to assess the possible involvement of adenosine receptors in the antioxidative effect evoked by beer, we tried to analyze the gene expression of these receptors during treatment. However, for long exposure time (24 h), we were unable to reliably detect adenosine receptors expression in C6 cells under our experimental conditions, probably because of the very low yield of total RNA extraction achieved after H_2_O_2_ treatment, whereas it could be determined in SH-SY5Y cells. Adenosine A_2A_ receptors gene expression in C6 cells was not modified by hydrogen peroxide, but the combination of LB with this compound significantly increased gene expression at 30 min of treatment with respect to the control and H_2_O_2_ values (*p* < 0.05, both) (Figure 6a). In the same way, A_2B_ gene expression was not affected by H_2_O_2_ exposure, but it was significantly increased by LB and H_2_O_2_ with respect to the control (*p* < 0.01) and H_2_O_2_ (*p* < 0.001) cases at 30 min of treatment (Figure 6b). However, the combination of DB with H_2_O_2_ significantly (*p* < 0.05) decreased A_3_ gene expression with respect to H_2_O_2_ treatment at 30 min (Figure 6c). In general, the gene expression profiles of A_2A_, A_2B_, and A_3_ after combined treatments of H_2_O_2_ and beer extracts were similar to those detected at 24 h in cells treated with the extracts alone (Figure 2a,c,e), confirming the absence of gene expression modulation by H_2_O_2_ in C6 cells at 30 min.

**Figure 7:**
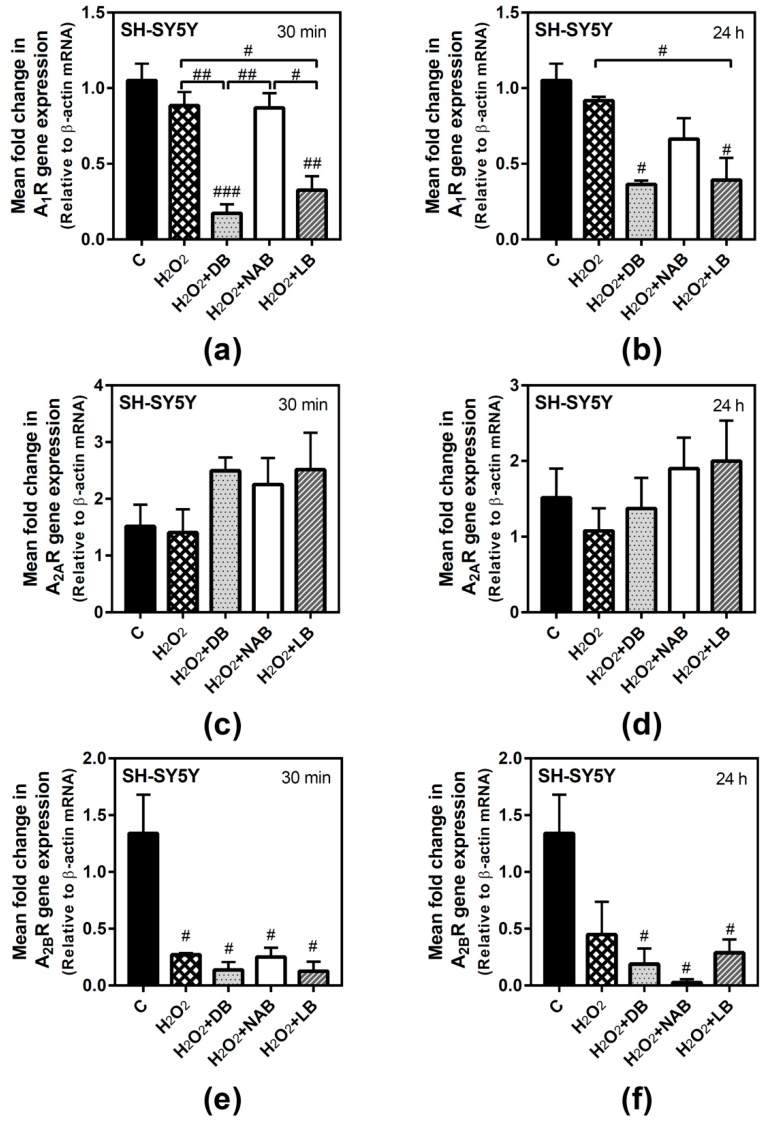

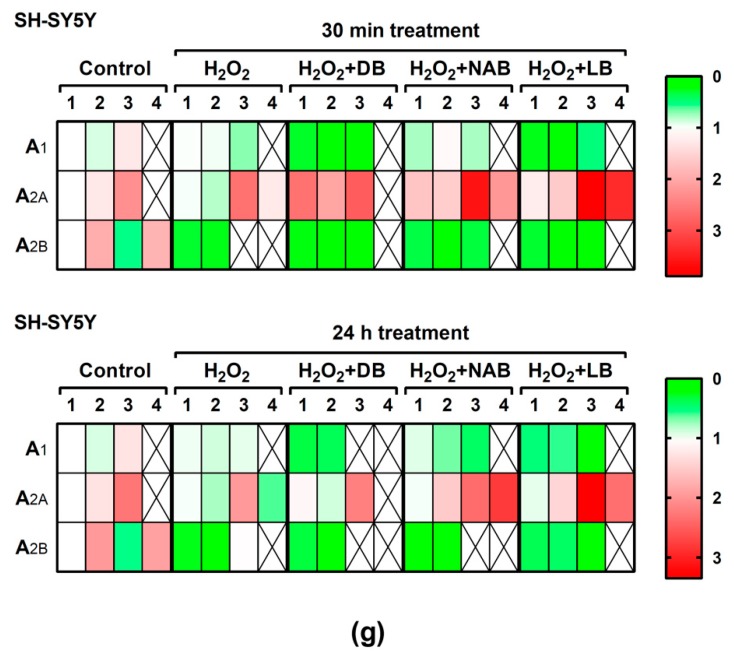
Adenosine receptors gene expression assayed by quantitative real-time RT-PCR in SH-SY5Y cells. After total RNA isolation, A_1_ (panels **a** and **b**), A_2A_ (panels **c** and **d**), and A_2B_ (panels **e** and **f**) gene expression levels were detected by TaqMan universal PCR following the protocol indicated in “Materials and Methods”. β-actin was used as an endogenous control in all assays. Treatments were performed for 30 min (**a**, **c**, **e**) and 24 h (**b**, **d**, **f**) with 50 µM hydrogen peroxide (H_2_O_2_) and DB, NAB, and LB extracts in SH-SY5Y cells. (**g**) Heatmap of gene expression levels obtained in the analyzed samples. Data are means ± SEM of, at least, three independent assays; # *p* < 0.05, ## *p* < 0.01, and ### *p* < 0.001, significantly different from control (C) or considered bars according to one-way ANOVA test.

In SH-SY5Y cells, adenosine receptors gene expression after H_2_O_2_ exposure was only significantly modified in the case of A_2B_ receptor gene, which was decreased at both times analyzed. A_1_ gene expression was significantly decreased by the combination of DB and H_2_O_2_ with respect to the control and oxidative stress conditions (*p* < 0.01 and *p* < 0.001, respectively) at 30 min of treatment (Figure 7a). At 24 h, A_1_ gene expression was significantly decreased by DB and LB with H_2_O_2_ treatment with respect to the control cases (*p* < 0.05), while H_2_O_2_ alone had no effect (Figure 7b). A_2A_ gene expression was not modified at 30 min (Figure 7c) or 24 h treatment (Figure 7d). In turn, A_2B_ gene expression was significantly decreased in all conditions assayed (*p* < 0.05, each one) at 30 min exposure (Figure 7e). Similar results were obtained at 24 h of treatment (*p* < 0.05, each one) with respect to the control cases (Figure 7f), although no significant differences were observed between treatment with H_2_O_2_ and treatment with H_2_O_2_ plus beer extracts.

## 4. Discussion

A moderate consumption of certain low-alcohol beverages, as wine and beer, could have beneficial properties for our health, showing a protective effect even on AD [47,48,49]. Oxidative stress is involved in neurodegenerative pathologies [50,51,52] and could be fought by the antioxidant ability of beer [5]. Our results show the different effects of beers (dark, non-alcoholic, and lager) in cells (C6 and SH-SY5Y) and the protection by these beers’ extracts against oxidative stress. The modulation of adenosine receptors gene expression and protein levels could be related to this antioxidative effect of beer.

Although several epidemiological studies indicate that the intake of certain low-alcohol beverages show beneficial effects, there is controversy about this. A frequent alcohol ingestion would increase dementia or cognitive decline as compared to a low-frequency intake [53,54,55,56]. Most of these studies involved wine or beer, and some of them associated a minor risk of dementia or AD with moderate consumption of wine [57,58,59]. These studies suggest polyphenols and alcohol as the possible responsible chemicals of these effects [60]. Thus, a moderate intake of beer would produce protective effects in the brain against plaques aggregation, associating these effects with the antioxidant properties of beer [16,17]. Other studies claim that a moderate consumption of beer increases silicon in our organism, reducing the absorption of aluminum in the digestive tract, a risk element in AD [61]. As these studies do not differentiate the effects due to alcohol from those due to other beer components, it could be suggested that the health benefits of this low-alcohol beverage probably could be related to its content in polyphenols and to its antioxidant properties.

Several authors postulate that beer ingestion protects from oxidative stress as a result of the high beer content of antioxidants [62,63,64,65]. Thus, these antioxidant compounds show protective effects in several experimental models. Rutin, a flavonoid glucoside present in several vegetables, is suggested as a multi-target preventive agent that may act as an adjuvant complementary molecule against oxidative stress in neuroblastoma IMR32 cells [66]. Geraniol has also showed a protective effect in SK-N-SH cells against oxidative stress induced by rotenone [67]. Other studies affirm that dietary flavonoids, specially flavonols, are associated with low rates of dementia in some countries [68].

Our results confirm the antioxidant ability of beer, as beer treatments (dark, non-alcoholic, and lager) at 24 h recovered cell viability in C6 and SH-SY5Y cells, avoiding cell death evoked by hydrogen peroxide. This property was reported for polyphenols present in wine, such as resveratrol, that was shown to inhibit cyclooxygenase 2 (COX-2) and decrease prostaglandin E_2_ (PGE_2_) in the presence of beta-amyloid peptide [69]. Moreover, procyanidins from seed grapes showed antioxidant benefits in PC-12 cells [70]. Our results show that dark beer is the treatment that presents the highest antioxidative effect, at 30 min and 24 h, and is able to produce the highest increase of adenosine A_1_ receptor protein levels in both cells models analyzed. The differences between the three kinds of beer may be due to their different phenolic composition. In fact, dark beer seems to have a higher amount of phenolic compounds than non-alcoholic and light beers [71]. Specifically, the levels of xanthohumol, isoxanthohumol, 8-prenylnaringenin, and iso-alpha-acids are higher in dark than non-alcohol and light beers, suggesting that the phenol B ring in hydroxylated flavonoids has higher antioxidant ability than other compounds [72]. Adenosine and inosine are also present in beers [73]. Adenosine exerts its effects through four specific adenosine receptors that are able to modulate neuronal and synaptic function. Adenosine A_1_ receptor is associated to neuroprotection by suppressing neural activity in presynaptic action, due to the decrease of excitatory neurotransmitters release [74], while A_2A_ receptor promotes neurotransmitters release and postsynaptic depolarization. In addition, adenosine receptors modify cell responses to neurotransmitters or to other receptor agonists [75]. Adenosine receptors have been involved in several neurodegenerative diseases. It is proved that adenosine receptors’ density change in AD patients [75]. For example, adenosine A_1_ and A_2A_ receptors were increased in the frontal cortex of AD patients [27]. Also, in the frontal cortex of Pick disease patients, adenosine A_1_ receptors were increased, while normal values were maintained in the occipital cortex [76]. A_2A_ receptors are also increased in the putamen of early PD [77]. Accordingly, antagonists of adenosine receptors have been related to protection and even treatment of AD and PD. Epidemiologic studies indicate that caffeine consumption, a non-selective antagonist of adenosine A_2A_ receptors, is associated with a low risk of development of AD, PD, and dementia, decreasing the cognitive decline of aging [38,78].

We must note that we worked with alcohol-free extracts of beer. In fact, cell viability was not altered by any of the beer extracts alone, confirming the absence of ethanol. Therefore, the modulation of adenosine receptors here reported could be associated with beer components other than ethanol [62]. Moreover, NAB extract modulated, in some occasions, receptor gene expression in the same way as the other extracts.

At protein level, all beer extracts increased A_1_ receptor levels, considered as “protective”, in C6 and SH-SY5Y cells. The quantification of A_2A_ receptor was only performed in SH-SY5Y, and non-alcoholic beer increased A_2A_ receptor gene expression. However, the gene expression results did not correspond to the protein levels in the cases of adenosine A_1_ receptor in both cell models and of A_2A_ receptor in SH-SY5Y. These differences between gene and protein expression have been described previously [37,79], suggesting different post-translational mechanisms responsible for these discrepancies.

We can suggest that the antioxidative protection of beer observed in our results is related to the increase in adenosine A_1_ receptor. Dark beer was the most potent antioxidant treatment in both cell types and induced the highest increase of adenosine A_1_ receptor protein levels. In agreement with this, endogenous adenosine is able to modulate oxidative stress via A_1_ receptors in ischemic heart [80]. In mice, this receptor decreased coronary reactive hyperemia counteracting hydrogen peroxide production mediated by A_2A_ receptors in the heart [81]. In addition, the activation of A_1_ receptors reduced reactive oxygen species in ventricular myocytes [82]. Adenosine receptors function and level have been involved in neuroprotective and antitumoral mechanisms in C6 cells. Thus, A_1_ and A_2A_ receptors were found to be modulated by hypoxia [34] and glutamate excitotoxicity [37] as an attempt to protect C6 cells against these toxic insults. In addition, adenosine receptors have been related to C6 cell death processes induced by several molecules. A_2A_ receptors participate in cordycepin-induced apoptosis, p53 activation, and caspase-7 and PARP cleavage, as all of these processes were blocked by A_2A_ antagonists and small-interference RNA (siRNA) knockdown of A_2A_ receptors [33]. A_3_ receptors activation in C6 cells causes apoptosis by reducing the expression of Bcl-2 [36] and also mediates the anti-proliferative action of alpha-bisabolol [83] and indomethacin [35].

Similarly, in SH-SY5Y cells, adenosine receptors have been related to neuroprotection. In fact, these cells have been used as a model to study the neuroprotective action of new partial agonists of A_1_ receptors [32]. A_2A_ receptor blockade also neuroprotects these cells against neurotoxicity induced by α-synuclein, which is involved in PD [41]. In addition, A_1_ and A_2A_ receptors have been also involved in neuroprotection against β-amyloid peptide neurotoxicity, which is related to AD [38,40], and in the neuroprotective action evoked by fullerene nanoparticles against hypoxic insult [39].

The three beers analyzed herein have different organoleptic properties and probably different composition and concentration of compounds such as polyphenols, vitamins, minerals, and nucleosides, as it has been demonstrated for other beers [13,71,72,73]. The effects of specific compounds have been studied in other models. Resveratrol is a polyphenol present in red wine and in beer. Some of resveratrol molecular targets are estrogen receptor α, cyclooxygenase 1, NF-ΚB, and adenosine receptors [23,24]. Previous studies from our group showed that this polyphenol is able to modulate adenosine-mediated signaling in SAMP8 mice, a model of AD, suggesting a neuroprotective role for resveratrol, acting through adenosine receptors, against neurodegenerative diseases [43]. Moreover, adenosine receptors seems to mediate in the anti-inflammatory activity of resveratrol in astrocytes, thus exerting an important role for resveratrol-mediated glioprotection [84]. In addition, tea compounds, such as polyphenols, teanine, caffeine, and teaflavines, antagonize adenosine A_2A_ receptors and exhibit neuroprotective and antioxidant properties, which could be related to decreased PD and AD risk by reduction of oxidative stress [85]. It seems clear that flavonoids have neuroprotective effects against aging in neurodegenerative diseases such as AD [68], and some of these compounds are present in beers.

Recently, we have reported that the polyphenol resveratrol acts as a non-selective agonist of adenosine receptors. Future research will be necessary to determine whether this or other antioxidant molecules present in beer, such as xanthohumol, are involved in the mechanism underlying adenosine receptors modulation. Also, future in vivo studies would be needed to determine the physiological effect of these antioxidant molecules in animals.

There are no studies, at least to our knowledge, about the effect of beer on adenosine receptors. This is the first time that beer extracts are reported to modulate these receptors, which are endogenously expressed in the neuronal and glial cell models used herein. In addition, this receptors’ modulation could be responsible for the protective effect of beer extracts against oxidative stress. Such receptor modulation and protective effect could be of relevance in AD, where oxidative stress and adenosine receptors have been involved.

## 5. Conclusions

In summary, this study showed that different beers (dark, non-alcoholic, and lager) have antioxidant protective properties, being dark beer the strongest antioxidant among them. These beers are also able to modulate adenosine receptors gene expression and protein levels, which could be involved, at least in part, in the protective effect of beer against oxidative stress elicited by hydrogen peroxide in two different (glioma and neuroblastoma) cell models.
